# The Medical News Visiting List for 1887

**Published:** 1886-11

**Authors:** 


**The Medical News Visiting List for 1887.** Philadelphia: Lea Brothers & Co.

Of the many visiting lists for the ensuing year, which will soon be issued, this is the first to reach us. We give it immediate notice because we think we will be doing our readers a service by advising them to examine the "Medical News" list before purchasing any other. It is very handsomely gotten up. The arrangement of its pages is, the writer thinks, excellent, but upon this point almost every physician has settled views of his own, dependent largely upon the particular style he has been in the habit of using. In our own opinion this is the most convenient, and altogether, the handsomest visiting list published.

